# Treatment of Indolent Ulcers by Vapor of Iodine

**Published:** 1860-01

**Authors:** 


4. Treatment of Indolent Ulcers by Vapor of Iodine. During the last three years nearly all the cases of indolent ulcers entered under our care to the U. S. Marine Hospital, have been treated by the vapor of iodine. The result is very satisfactory in nearly all cases; more so, by far, than that obtained by any other single method. Its advantages are conceived to be these:

1. Cleanliness and facility of application.
2. Rapidity of cicatrization.
3. Destruction of the odor of the ulcer. Iodine acts as a disinfectant, like chlorine.

The manner of using it is as follows:

1. Dress the ulcer with simple create, spread on lint.
2. Take from one to four grains of iodine, according to the size and degree of indolence of the ulcer, folded in several layers of lint, and place it on the ulcer, over the first layer.
3. Cover this with a piece of oiled silk and tin foil, which should be large enough to extend beyond the edges of the ulcer. This is to prevent rapid vaporization, and it should be secured by a roller.

The warmth of the member speedily vaporizes the iodine, and a sensation of warmth is perceived by the patient on the ulcerated surface. If applied in too large quantity, or too directly on the surface, the Iodine acts as an escharotic. Care is therefore required in this respect.

